# Effects of ethanolic extract of green tea on decreasing the level of lipid profile in rat

**Published:** 2013

**Authors:** Farjad Amanolahi, Hasan Rakhshande

**Affiliations:** 1 *Department of Pharmacology and Medicinal plant Research Center, School of Medicine, Mashhad University of Medical Science, Mashhad, I. R. Iran*

**Keywords:** Anti-hyperlipidemic, Aqueous Extract, Cholesterol, Green Tea, Nicotinic Acid

## Abstract

**Objective:** The aim of the present study was to elucidate the possible biochemical improving effect in lipid metabolic that may result from continuous treatment with Green Tea extract in normal albino rats and those rendered hyperlipidemic by long term supplementation of fat-enriched diet.

**Materials and Methods:** Fifty male albino rats aged six weeks with 200±10 g weight were randomly divided into five groups: Group A (negative control), groups B (positive control), Group C (treatment with drug), Group D (treatment with extract), and Group E (prevention with extract). All groups except Group A were received fat-enriched diet throughout the study. Group C received 25 mg/kg/day of the nicotinic acid from day 28 to the end of study. Group D was also treated with 100 mg/kg/day of the extract from day 28 to the end of study and finally group E was also treated with 100 mg/kg/day of the extract from the start of the study to the end. Lipid levels were determined weekly. Data were expressed as mean±SEM which were calculated using SigmaPlot® software. The obtained data were statistically analyzed using Student's t–test.

**Results:** In group (D), total cholesterol, LDLc, HDLc, and triglyceride levels were significantly decreased by 33.3%, 30.2%, 40%, respectively, compared with the group C (p<0.001 ). There was no significant difference in lipid profile in group E through the study. Nicotinic acid in group C decreased the serum levels of all measured parameters (p<0.001). The body weight of animals in treated groups with extract (groups D & E) were decreased by 8% (p<0.01).

**Conclusion:** The results of this study demonstrated that the extract of green tea has a hyperlipidemic lowering effect.

## Introduction

Hyperlipidemia, resulting from lipid metabolic changes, is a major cause of cardiovascular disturbances (Chobanian, 1991 ▶). Although a high level of serum cholesterol is identified as a risk factor for atherosclerosis and coronary heart disease (Kanel et al.,1971 ▶), the role of a high level of triglycerides has only recently been established as an independent risk factor for such diseases (Cambien et al., 1986 ▶; Austin, 1989 ▶). The therapeutic target in such conditions is to lower blood and tissue levels of cholesterol and lipids. Currently, available antihyperlipidemic drugs are always associated with some side effects such as gastric irritation, nausea, diarrhea, hyperuricemia, myositis, flushing, dry skin, and abnormal liver function (Kumar et al., 2008 ▶). 

Nutriceuticals or nutrients with health impacts constitutes, are a considerable area of interest in minds of patients, physicians, and researchers because of their safety and availability. Green tea, derived from the plant *Camellia sinensis* is the most popular beverage in Japan and China and is cultivated in more than 30 countries. Tea is among drinks with health potentials at different aspects and its importance comes from its use on a daily basis by the young and the old in all countries (Fujita et al., 2000 ▶; Fujita et al., 2001 ▶). *Camellia sinensis* extract (Black tea) contains major groups of active principles including theaflavins, thearubigins, catechins, and flavones. Some reports showed a relationship between some types of tea and some lipid parameters including cholesterol and some studies showed an apparent protective role against cardiovascular disease or stroke with high intakes of black tea or flavonoids (Keli et al., 1996 ▶; Geleijnse et al., 1999 ▶) as well as the susceptibility of LDL to oxidation (Ishikawa et al., 1997 ▶). A number of studies have shown green and black teas exert anticholesterolemic effect (Kono et all., 1992 ▶; Muramatsu et al., 1986 (b)) ▶. Epidemiological studies found consumption of green or black tea was inversely correlated with plasma cholesterol concentrations (Kono et all., 1992 ▶; Stensvold et al., 1992 ▶). Maramatsu et al. (Maramatsu et al., 1986 (a) ▶) suggests that addition of 1–2% green tea extract to the lard/cholesterol diet reduced blood cholesterol levels in rats. Therefore, the aim of the present study was to discover if there is a similar anti-hyperlipidemia effect in green tea which grows in the North of Iran. Therefore, we designed this research to assess the hyperlipidemia improving profile of green tea as a nutriceutical approach to hyperlipidemia in rats rendered hyperlipidemic by long term feeding on high fat diet.

## Material and methods

**Preparing of green tea extract**

The green tea was prepared from the North of Iran, Gillan. The method used was based on the method of Huang et al., 1992. Briefly, In Digestion method, 250 g dry and powder of green tea was mixed with 1000 ml ethanol 95^° ^in 35 °C. The door of extracting vessel was closed and the solution was kept at this temperature using a heater. After 24 h and elimination of the solvent, 33.4 g dry green tea remained.

**Animal study**

Fifty male albino rats aging 6 weeks of approximate weights 200±10 g were used in this study. Rats were kept in separate cages and allowed to a plenty of water and food at room temperature. After one week of acclimatization, they received different treatments as Group A (rats were fed on normal diet and received no drugs; kept as negative control), Group B (rats were fed on fat-enriched diet and received no drug, kept as positive control for all experimental groups). Group C (rats were fed on fat-enriched diet and received 25 mg/kg/day of the nicotinic acid from day 28 to the end of the study, kept as the standard treated group), Group D (rats were fed on fat-enriched diet and received 100 mg/kg/day of the extract from day 28 to the end of the study, kept as positive treated group), Group E (rats were fed on fat-enriched diet and received 100 mg/kg/day of the extract from the start of the study to the end, kept as concurrent treatment group).

 The macro-nutrient composition of the diet was as follows (g/100g anhydrous mix): protein 21, fiber 8, fat 18, and carbohydrate 41. The diet also contained 2 wt% cholesterol, 1 wt% sodium cholate, and a standard vitamin and mineral mix, prepared according to rat nutritional requirements. The main fats consisted of coconut kernel (18 wt%; 12% fat), coconut oil (2.5 wt%), and corn oil (2.5 wt%) ( Maramatsu et al., 1986 (a) ▶).

**Sampling**

Individual body weight was recorded twice a week and food consumption was registered once a week for each group. At the end of each week, blood for serum was collected. Samples were collected from the venous plexus located at the medial canthus of the eye by means of heparinized capillary tubes. The collected blood was allowed to clot at room temperature for an hour and then refrigerated for another hour for clot retraction. 

Clear sera were separated by centrifugation at 3000 rpm for 10 minutes and then collected in Eppendrof’s tubes using automatic pipettes. Serum samples were kept in deep freezer (-20 °C) for analysis of the biochemical parameters including total lipids (TL), total cholesterol (TC), triglycerides (TGs), high density lipoprotein-cholesterol (HDLc), and low density lipoprotein-cholesterol (LDLc) that were determined enzymatically on a COBAS FARA analyzer (Roche Diagnostics, Switzerland)

**Statistical analysis**

Data were expressed as mean±SEM which were calculated using SigmaPlot® software. The obtained data were statistically analyzed using Student's t–test to express the differences between groups according to Snedecor and Cokran (1980).

## Results

**Levels of Ch, TG, HDL and HDL**

As shown in Tables 1, 2, 3, and 4, there were significant increases in cholesterol, triglycerides, LDLc, and HDLc concentrations in rats fed on fat-enriched diet, compared with rats received basal diet (p<0.05 at the end of 4^th^ week and p<0.01 at the end of study). While administration of green tea extract to normal rats caused insignificant changes in these parameters all over the period of the experiment, its administration significantly decreased their serum levels in rats fed on fat-enriched diet. At the end of the 4^th^ week of experiment, the level of lipid profile (Ch, TG, HDLc, and LDLc) have a significant increase with a minimum value of p<0.05 and maximum value of p<0.001.

From the beginning of the 5^th^ week to the end of the study, consumption of extract was resulted in a meaningful reduction in cholesterol, TG, LDLc, and HDLc levels (Tables 1, 2, 3, and 4). Moreover, a significant reduction in lipid profile in group C was seen that was a result of nicotinic acid (p<0.01). Except for Group E that have a significant decrease in TG and LDLc, there was no significant difference in lipid profile in group E throughout the study that indicates the preventive effect of extract in lipid plasma levels. On the other hand, this result shows that using it have a good potency to produce a stability stage in lipid profile.

**Data of measureing animals** **weight**

Data show that consumtion of grean tea extract have significant decrease in the weight of rats in groups C (-10%), D (-8%) and E (-8.3%) compare of group B (+21%). p<0.001

**Table 1:** Effect of green tea extract on serum cholesterol. Effect of oral administration of 100 mg/kg/day for 56 days on serum cholesterol concentration (mean±SEM; mg/dl) in albino rats fed on basal and fat-enriched diets (n=10).

	**Group A**	**Group B**	**Group C**	**Group D**	**Group E**
**Weak 1**	60 ± 1.5	68 ± 1.5	65 ± 1.2	61 ± 1.9	61 ± 1.2
**Weak 2**	64 ± 2	73 ±2.4	74 ± 2.1	68 ± 1.2	68 ± 1.08
**Weak 3**	62 ± 2	84 ± 2	83 ± 1.4	79 ±1.3	72 ± 1.1
**Weak 4**	61 ± 2	92 ± 2.1*	96 ± 1.6*	92 ± 1.1*	69 ±1.3
**Weak 5**	62 ± 1.5	98 ± 1.4	94 ± 1.1	85 ± 1.4	71 ± 1.1
**Weak 6**	61 ± 2	110 ± 1.5	82 ± 2	80 ± 1.1	63 ± 1.1
**Weak 7**	60 ± 2	119 ± 1.3	73 ± 1.6	71 ± 1.3	62 ± 1.4
**Weak 8**	62 ± 2	128 ± 1.1**	60 ± 1.1**	65 ± 1.1**	60 ± 1

Group A: Negetive control; Group B: Positive control; Group C: treated with nicotinic acid 25 mg/kg/day; Group D: treated with extract 100 mg/kg/day; Group E: treated as prevention.

* p<0.05;

** p<0.01.

**Table 2:** Effect of green tea extract on serum TG. Effect of oral administration of 100 mg/kg/day for 56 days on serum cholesterol concentration (mean±SEM; mg/dl) in albino rats fed on basal and fat-enriched diets (n=10).

	**Group A**	**Group B**	**Group C**	**Group D**	**Group E**
**Weak 1**	75 ± 1.2	75 ± 1.1	78 ± 1.1	79 ± 1.1	78 ± 1.1
**Weak 2**	73 ± 1.4	78 ± 1.2	89 ± 1.5	89 ± 1.6	85 ± 1.8
**Weak 3**	75 ±1.07	80 ± 1.7	96 ± 1.1	103 ± 1.3	85 ± 1.5
**Weak 4**	78 ± 1.1	91 ± 1.5*	105 ± 1.2**	115 ± 1.1***	85 ± 1.5
**Weak 5**	80 ± 1	98 ± 1.1	100 ± 1.1	110 ± 1.1	78 ± 1.1
**Weak 6**	76 ± 1	109 ± 1.2	86 ± 1	91 ± 1.4	74 ± 1.1
**Weak 7**	73 ± 1.1	120 ± 1.1	74 ± 1.5	86 ± 1.2	71 ± 1.4
**Weak 8**	75 ± 1.2	125 ± 1.02***	70 ± 1.1**	76 ± 1.5***	68 ± 1.1*

Group A: Negetive control; Group B: Positive control; Group C: treated with nicotinic acid 25 mg/kg/day; Group D: treated with extract 100 mg/kg/day; Group E: treated as prevention.

* p<0.05;

** p<0.01;

*** p<0.001.

**Table 3:** Effect of green tea extract on serum LDLc. Effect of oral administration of 100 mg/kg/day for 56 days on serum cholesterol concentration (mean±SEM; mg/dl) in albino rats fed on basal and fat-enriched diets (n=10).

	**Group A**	**Group B**	**Group C**	**Group D**	**Group E**
**Weak 1**	32 ± 1.2	30 ± 1.5	29 ± 1.4	31 ± 1.1	33 ± 1.2
**Weak 2**	31 ± 1.4	33 ± 1.1	36 ± 1.1	35 ± 1.2	33 ± 1.1
**Weak 3**	32 ± 1.1	37 ± 1.1	44 ± 1.2	44 ± 1.0	35 ± 1.3
**Weak 4**	32 ± 1.1	41 ± 1.1*	50 ± 1.0**	50 ± 1.1**	38 ± 1.1
**Weak 5**	30 ± 1.2	48 ± 1.3	44 ± 1.1	47 ± 1.1	35 ± 1.4
**Weak 6**	29 ± 1.1	52 ± 1.2	40 ± 1.2	40 ± 1.2	31 ± 1.1
**Weak 7**	33 ± 1.2	54 ± 1.1	36 ± 1.1	35 ± 1.2	29 ± 1.1
**Weak 8**	32 ± 1.4	60 ± 1.1**	28 ± 1.1**	29 ± 1.1**	29 ± 1.1*

Group A: Negetive control; Group B: Positive control; Group C: treated with nicotinic Acid25mg/lg/day; Group D: treated with extract 100 mg/kg/day; Group E: treated as prevention.

* p<0.05;

** p<0.01.

**Table 4:** Effect of green tea extract on serum HDLc. Effect of oral administration of 100 mg/kg/day for 56 days on serum cholesterol concentration (mean±SEM; mg/dl) in albino rats fed on basal and fat-enriched diets (n=10).

	**Group A**	**Group B**	**Group C**	**Group D**	**Group E**
**Weak 1**	32 ± 1.2	32 ± 1.1	32 ± 1.3	32 ± 1.1	32 ± 1.1
**Weak 2**	34 ± 1.1	34 ± 1.3	36 ± 1.2	37 ± 1.6	36 ± 1.1
**Weak 3**	31 ± 1.3	32 ± 1.1	41 ± 1.6	37 ± 1.1	41 ± 1.2
**Weak 4**	30 ± 1.02	34 ± 1.3	48 ± 1.4*	41 ± 1.5*	38 ± 1.1
**Weak 5**	28 ± 1.1	38 ± 1.5	47 ± 1.6	40 ± 1.2	37 ± 1.1
**Weak 6**	32 ± 1.1	41 ± 1.5	40 ± 1.3	34 ± 1.1	35 ± 1.5
**Weak 7**	32 ± 1.1	44 ± 1.6	37 ± 1.2	30 ± 1.1	31 ± 1.1
**Weak 8**	33 ± 1.5	52 ± 1.4**	31 ± 1.1**	27 ± 1.1**	28 ± 1.1

Group A: Negetive control; Group B: Positive control; Group C: treated with nicotinic acid 25 mg/kg/day Group D: treated with extract 100 mg/kg/day; Group E: treated as prevention.

* p<0.05;

** p<0.01.

## Discussion

Improper lipid intake is known to be related to a range of serious diseases and disabilities in many animals (Rizvi et al., 2003 ▶; Guermani-Nicolle et al., 2001 ▶) including mammals (Dalessandri et al., 1995 ▶). Consumption of excessive quantities or improper types of lipids may cause hyperlipidemia (Oliveros et al., 2003 ▶). Peroxidation of lipids is also related to coronary heart disease (Hertog et al., 1993 ▶). 

Treatment of hyperlipidemia may be with therapeutic medicines or through natural edible materials which help to lower serum lipid levels. Natural edible materials have the advantage in that they avoid side effects often associated with medications, while still improving or healing the hyperlipidemia (Yoon et al., 2003 ▶). Many natural edible materials (or their components) have been identified as having antihyperlipidemic effects. Data of the present study revealed that hyperlipidemia, induced by continuous supplementation of high fat (coconut oil 2% w/w) and high cholesterol (1% w/w) diet caused marked alterations (mainly increase except HDLc which is decreased) in almost all lipid parameters of rat groups fed on such diet. Moreover, the obtained hyperlipidemia was associated with elevated markers for some organ dysfunction such as liver, kidneys, heart, and aorta together with considerable histopathological changes in these organs (data not shown). These findings led us to use such rats as a model for hyperlipidemia to assess the possible antihyperlipidemic role of iced black tea that is a popular drink liked by almost all people all over the world. 

Data of the present study (Table 1) demonstrate a significant increase in serum total cholesterol concentration of rats fed on fat-enriched diet all over the two-month period of the experiment, compared with the control rats that received basal diet. This result is consistent with those achieved by (Diaz et al., 2000) who reported that rabbits fed with the atherogenic diet showed marked increase in plasma total cholesterol. 

The result is also consistent with that of (Abdel-Maksod et al., 2002) who reported that mice and rats received cholesterol-enriched diet showed sever hypercholesterolemia, elevated plasma serum LDLc and VLDLc compared with those fed a normal diet. In addition, (Hammad, 2002) reported that, administration of laying hens with diet rich in cholesterol diet led to marked elevation in plasma total cholesterol. Rise in serum cholesterol might be attributed to the reduced catabolic rate of serum TC or reduced activity of hepatic cholesterol-7-alpha-hydroxylase, the rate limiting enzyme in bile acid synthesis from cholesterol (Abdel-Maksod et al., 2002). Moreover, the rise in serum TC observed in this study could be attributed to increased HMG-CoA reductase activity in the liver of animals fed on fat-enriched diet and the reduced rate of the clearance of LDL from circulation due to defective LDL receptors associated with increase of plasma TC concentration (Zulet et al., 1999).

Data of the present study demonstrated in Table 2 show a significant increase in triglycerides in animals kept on fat-enriched diet compared with their corresponding control. This result may be in accordance with those recorded by Margaret, et al., 2000, who found that a hyperlipidemic diet caused a significant increase in the level of the plasma triacylglycerols and an increased content of cholesterol in the liver, despite the fact that the diet produced a cessation of endogenous cholesterol synthesis. Such significant rise in serum triacylglycerols level may be attributed to the decrease of activity of lipase which is an insulin-dependent enzyme involved in triglyceride clearance from plasma by mediating triglyceride lipolysis into glycerol and FFA (Yost et al., 1995). 

Data obtained in the present study that are demonstrated in Tables 3 and 4 revealed significant increases in serum LDLc and HDLc in the group of rats that was fed on fat-enriched diet all over the period of the experiment, compared with the corresponding control group. These results are in accordance with those reported by Abdel-Maksod et al., 2002, who reported that mice and rats receiving cholesterol-enriched diet showed severe elevated plasma LDLc compared with those kept on a normal diet. Hussein and colleagues concluded that the elevated serum LDLc seemed to be related mainly to reduced catabolic rate that occurs when the production of LDL exceeds the capacity of LDL receptors present on hepatocytes, in other words, when the efflux of cholesterol from the liver becomes more than its influx. Mahley and Habcombe, 1977, stated that both dietary fat and cholesterol may change the lipoprotein content of serum and affect the different classes of lipoproteins, LDL, and HDL.

In Tables 1, 2, 3, and 4 we can see a good effect of drug and extract in the end of the study compared with the 4^th^ week. In Table 1, after 28 days from the start of the study, the concentration of serum total cholesterol have a significant increase compared with the start of the experiment (p<0.05). After addition drug (25 mg/kg/day) in group C and extract (100 mg/kg/day) in group D, we can see a significant decrease in cholesterol concentration (p<0.01). A similar effect was seen in other profile of lipid such as TG, LDLc, and HDLc. (Tables 2, 3, and 4).

Data of cholesterolp, TGp, HDLc and LDLc in group 5 was analyzed with sigma plot and showed normality in the data. It means that there was not significant difference between data. Actually it means that consumption of the extract produced a steady stage in serum total lipid profile and addition of the extract may have a prevention effect and it stopped the increasing in lipid profile (Table 2, group 5).

The comparison among positive control group, therapy group, and prevention indicate the antihyperlipidemic property of the green tea extract. Today many different chemical medicines are used to control blood lipid and decrease the risk factors of heart and blood vessel illnesses which each one has its undesirable effects. Based on this study, daily consumption of green tea which is brewed up accompanied by a desired diet will have a significant role in decreasing the risk of counteracting illnesses with high levels of blood lipid.
